# Effects of energy drinks on myogenic differentiation of murine C2C12 myoblasts

**DOI:** 10.1038/s41598-023-35338-7

**Published:** 2023-05-25

**Authors:** Sun Young Park, Georgia Karantenislis, Hannah T. Rosen, Hong Sun

**Affiliations:** 1 Division of Environmental Medicine, NYU Grossman School of Medicine, 341 East 25 Street, New York, NY 10010 USA; 2 John F. Kennedy High School, Bellmore, NY USA

**Keywords:** Developmental biology, Environmental sciences

## Abstract

Energy drinks, often advertised as dietary supplements that enhance physical and mental performance, have gained increasing popularity among adolescents and athletes. Several studies on individual ingredients such as caffeine or taurine have reported either adverse or favorable influences on myogenic differentiation, a key process in muscle regeneration to repair microtears after an intense workout session. However, the impact of different energy drinks with various formulas on muscle differentiation has never been reported. This study aims to examine the in vitro effects of various energy drink brands on myogenic differentiation. Murine C2C12 myoblast cells were induced to differentiate into myotubes in the presence of one of eight energy drinks at varying dilutions. A dose-dependent inhibition of myotube formation was observed for all energy drinks, supported by reduced percentage of MHC-positive nuclei and fusion index. Moreover, expression of myogenic regulatory factor MyoG and differentiation marker MCK were also decreased. Furthermore, given the variation in formulas of different energy drinks, there were remarkable differences in the differentiation and fusion of myotubes between energy drinks. This is the first study to investigate the impact of various energy drinks on myogenic differentiation and our results suggest an inhibitory effect of energy drinks in muscle regeneration.

## Introduction

Energy drinks are beverages marketed to improve overall mental and physical performance. The global energy drink market has grown exponentially in the past couple decades, and is forecasted to grow from $53.01 billion in 2018 to $86.01 billion by 2026^1^. According to the National Center for Complementary and Integrative Health, men between the ages of 18–34 consume the most energy drinks and one in three adolescents drink them regularly^2^. Since its first introduction to the U.S. market in 1997, there has been a significant increase in energy drink consumption due to aggressive marketing strategies targeting athletes and adolescents^3,4^. This is a concern because emerging studies show that certain ingredients in energy drinks are associated with adverse cardiovascular, neurological, gastrointestinal, metabolic, dental, and renal effects^3,5^. A majority of energy drinks contain similar ingredients including caffeine, sugar, vitamins (i.e. vitamin B2, B3, B6, and B12), and non-nutritive stimulants (i.e. taurine, L-carnitine, D-glucuronolactone, inositol, guarana extract, ginseng, and yerba mate)^5^.

Most of the ingredients in energy drinks are readily available to consumers so the beverages are not subject to regulation by the US Food and Drug Administration (FDA)^6^. Caffeine (an adenosine receptor antagonist) is a stimulant that can activate neuronal control pathways in the central as well as the peripheral nervous system^6^. Excessive caffeine consumption, above the FDA recommended limit of 400 mg/day, is associated with a greater risk for insomnia, agitation, anxiety, and gastrointestinal disorders^7^. Taurine (an amino sulfonic acid) is an organic compound found extensively in animal tissues and is believed to have multiple beneficial effects such as promoting cognitive functions and regulating skeletal muscle contractile functions^6,8^. A recent study suggests that taurine may have antioxidant properties for athletes such as attenuation of oxidative damage in muscles upon intensive exercise^9^. Glucuronolactone (a naturally occurring substance generated in small amounts in the body) is added to energy drinks to increase mental performance but little research has explored the efficacy of this food additive^6^. Emerging research suggests that food additives in energy drinks (i.e. glucuronolactone, gluconolactone, and taurine) can have neurotoxic effects^10^. Energy drinks also contain large amounts of sugar in which overconsumption is well understood to be associated with adverse side effects like irregular heartbeat, irregular blood pressure, and obesity^11–13^. Despite increasing evidence that some ingredients in energy drinks are associated with adverse health outcomes, the effects of energy drink consumption on the health and wellness of adolescents and athletes are still largely unknown.

Myogenesis or myogenic differentiation is the process in which myoblasts undergo irreversible cell cycle arrest to fuse into multinucleated myotubes, accompanied by a gradual increase in muscle specific gene expression^14,15^. Muscle development in vertebrates can be divided into two stages—embryonic and adult myogenesis. In embryonic myogenesis, skeletal muscles are derived from somites that divide into the ventral mesenchymal sclerotome and the dorsal epithelia dermomyotome^16^. The epaxial and hypaxial lips of the dermomyotome have myogenic precursor cells that undergo epithelial-mesenchymal transition (EMT) to differentiate and elongate into myotome myocytes^16^. The epaxial myotome generates deep back muscles and the hypaxial myotome generates body wall muscles as well as fore- and hind-limb muscles^16^. Additionally, the unsegmented paraxial mesoderm generates craniofacial skeletal muscles which can be divided into the four categories: extra-ocular, laryngoglossal, branchial, and axial neck muscles^16^. In adult myogenesis, satellite cells are reactivated for maintenance and repair of muscle tissues^16,17^. Satellite cells are a stem-cell population essential for repairing skeletal muscles and express a transcription factor Pax7 in their quiescent state^16^. Embryonic and adult myogenesis are similar in that the differentiation of skeletal muscles is orchestrated by a core network consisting of myogenic regulatory factors (MRFs) including myoblast determination protein 1 (MyoD), myogenin (MyoG), myogenic factor 6 (also known as Mrf4), and myogenic factor 5 (Myf5)^15,16,18^. Skeletal muscles are tissues consisting of multiple multinucleated fibers that constantly undergo remodeling in response to the body’s varying metabolic and functional demands^19^. Eccentric or lengthening contractions as a result of exercise may lead to muscle injury, which elicits muscle regenerative responses^19,20^. Upon muscle injury, a pool of skeletal muscle stem cells—also referred to as satellite cells—are activated to migrate, proliferate, and differentiate into new myotubes that are responsible for repairing injured muscle fibers^16,20,21^. A subset of the satellite cells will return to a quiescence state until subsequent muscle injury^20^. Muscle regeneration is generally initiated in the first 4–5 days post injury and fully repaired in the following 3–4 weeks^22^. A plethora of recent studies indicate that there is a high prevalence of energy drink consumption among athletes and adolescents worldwide^23–25^, which is a concern because much of the adverse health effects associated with energy drinks are not well understood. This is a growing public health concern because energy drink consumption has been associated with fatal outcomes related to the cardiovascular system such as myocardial infarctions, cardiomyopathies, and sudden cardiac death^26^. Given the high consumption rate among athletes and adolescents, it is critical to understand the influence of energy drinks on the muscle regeneration process after microtears from intensive workouts.

This study was conducted to determine and compare the effect of in vitro exposures to a variety of energy drink formulations on myogenic differentiation. Mouse C2C12 myoblast cells, a well-established model used to study muscle differentiation and regeneration^27^, were exposed to eight energy drinks from four major brands in the market: RedBull, RedBull Zero, Monster Energy, Monster Ultra Paradise, Rockstar, Rockstar Sugar Free, Celsius Live Fit, and Celsius Heat. Our results revealed that all energy drinks displayed a dose-dependent inhibition on myotube formation. These results will provide further insight on the effects of energy drinks on skeletal muscles.

## Results

### Energy drink ingredients

To compare the ingredients of leading energy drink brands, we selected the following energy drinks for this study: RedBull, RedBull Zero, Monster Energy, Monster Ultra Paradise, Rockstar, Rockstar Sugar Free, Celsius Live Fit, and Celsius Heat. The individual ingredients and their amounts for all energy drinks are listed in Table 1. All ingredient concentrations are normalized to a volume of 100 ml. According to Table 1, some common ingredients across all energy drinks include caffeine, carbohydrates, niacin (vitamin B3), vitamin B6, and vitamin B12. Celsius Heat has the highest concentration of caffeine while Monster Ultra Paradise has the lowest concentration. Compared to other energy drinks, Celsius Live Fit and Celsius Heat are unique because they contain vitamin C and chromium chelate. Additionally, Celsius Heat is the only drink that contains l-citrulline. There are also interesting differences between energy drinks sold from the same brand. Compared to RedBull Zero, RedBull contains higher concentrations of sodium, carbohydrate, and total sugars. Compared to Monster Ultra Paradise, Monster Energy contains higher concentrations of caffeine, sodium, carbohydrate, and total sugars. Rockstar contains a higher concentration of carbohydrates and total sugars but Rockstar Sugar Free contains a significantly higher concentration of sodium. Compared to Celsius Heat, Celsius Live Fit contains higher concentrations of carbohydrates, calcium, vitamin Bs, vitamin C, and chromium chelate but Celsius Heat has a higher concentration of caffeine. Although a majority of energy drink formulations consist of a combination of caffeine, taurine, sugars, and vitamins, it is evident that there are differences in the amount of each ingredient added into the drinks.

**Table 1:** Energy drink ingredient table.

	RedBull	RedBull Zero	Monster Energy	Monster Ultra	Rockstar	Rockstar Sugar Free	Celsius Live Fit	Celsius Heat
Caffeine (mg)	32	32	33.827	29.598	33.827	33.827	56.338	63.425
Total fat (g)	0	0	0	0	0	0	–	–
Sodium (g)	42	12	78.224	65.539	14.799	50.740	0	0
Carbohydrate (g)	11.6	0.4	12.262	0.634	13.319	0.211	0.563	0.423
Total sugars (g)	10.8	0	11.416	0	13.319	0	0	–
Added Sugars (g)	10.8	0	11.416	0	13.319	0	–	–
Protein (g)	0	0	0	0	0	0	–	–
Calcium (mg)	12	12	–	–	–	–	14.085	10.571
Riboflavin or B2 (mg)	–	–	0.715	–	0.275	0.275	0.479	0.359
Niacin or B3 (mg)	6.4	6.4	8.457	8.457	3.383	3.383	5.634	4.228
Pantothenic acid or B5 (mg)	1.0	1.0	–	4.228	1.057	1.057	2.817	2.114
Vitamin B6 (mg)	1.7	1.7	0.863	0.863	0.359	0.359	0.563	0.423
Vitamin B12 (µg)	0.768	0.768	2.537	2.386	0.507	0.507	1.690	1.268
Vitamin C (mg)	–	–	–	–	–	–	16.901	12.685
Chromium chelate (µg)	–	–	–	–	–	–	14.085	10.571
l-Citrulline (g)	–	–	–	–	–	–	–	0.423
Original pH	3.4	2.93	2.82	3.20	3.45	3.47	2.98	2.90
Adjusted pH	7.18	7.17	7.19	7.23	7.17	7.15	7.18	7.13

### C2C12 cytotoxicity response to energy drinks

**Figure 1:**
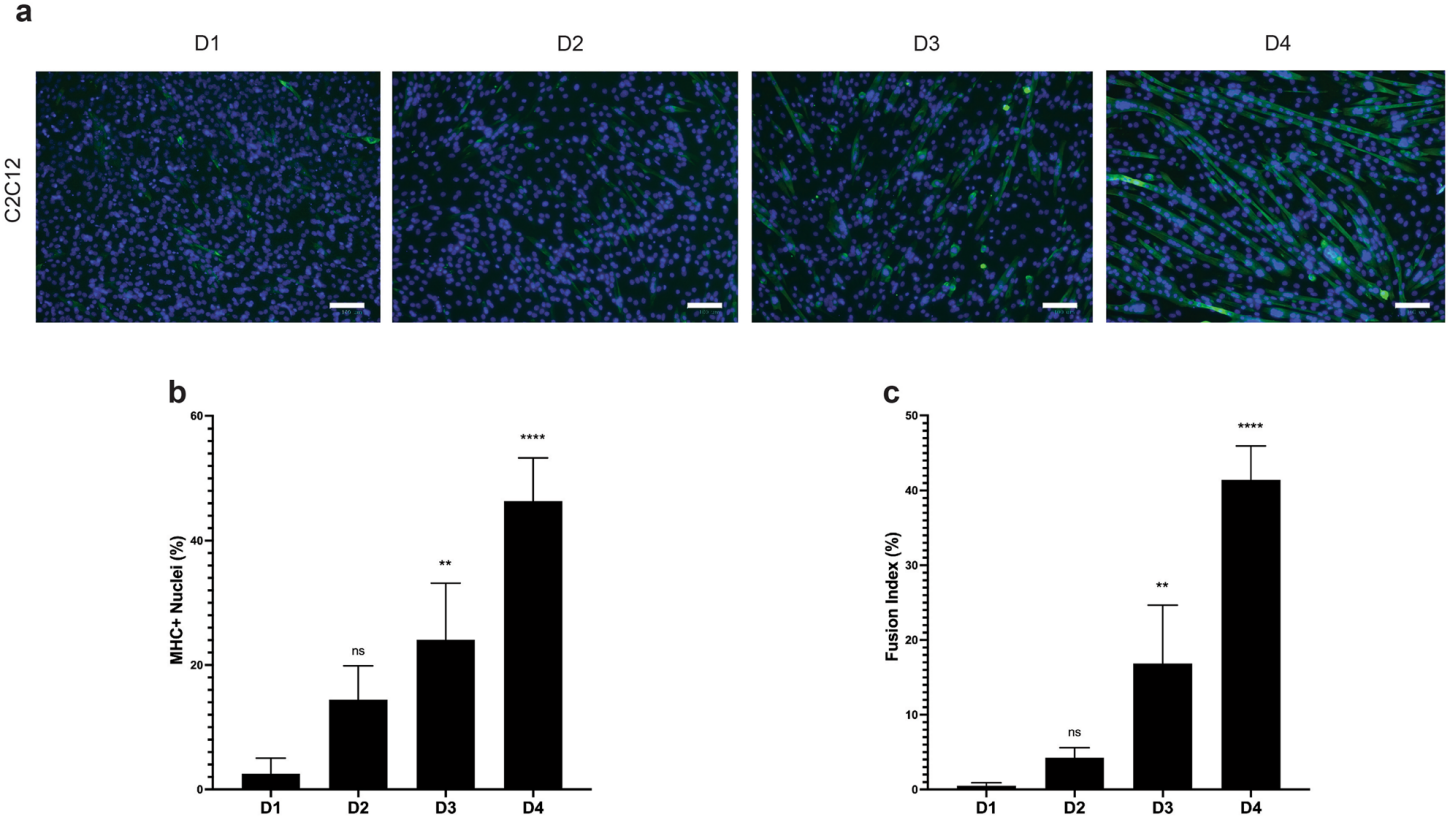
In vitro myogenic differentiation of C2C12 myoblasts. (**a**) Representative images of C2C12 cells were cultured in differentiation medium (DMEM + 2% horse serum + 1% penicillin–streptomycin) with no energy drinks for 4 days. From differentiation day 1 to day 4, cells were assessed for MHC + nuclei (%) and fusion index (%) through immunofluorescence (IF) staining. Differentiated myotubes were stained with MF20 (green) and nuclei were stained with DAPI (blue). Images are representative of two different experiments. Scale bar = 100 µm. (**b**) Quantification of the percentage of MHC + nuclei representative of differentiated myocytes in the absence of energy drinks. Bars are presented as means ± SD. (**C**) Quantification of the fusion index representative of the percentage of myocyte fusion in the absence of energy drinks. Bars are presented as means ± SD. All significance compared to differentiation day 1 in panels (**b**) and (**c**) were evaluated using a one-way ANOVA test (*p < 0.05, **p < 0.005, ***p < 0.001, ****p < 0.0001).

To establish an in vitro myogenic differentiation system, C2C12 myoblasts were induced to differentiate in the absence of an energy drink for four days. Cell differentiation was evaluated daily by expression of myosin heavy chain (MF20) and formation of multinucleated myotubes (Fig. 1a). Myogenic differentiation was quantified as the percentage of MHC + nuclei out of the total number of nuclei. Myoblast fusion—a crucial step to form multinucleated myotubes—was assessed by the fusion index representative of the percentage of 2 or more MHC + nuclei in a single myotube out of the total number of nuclei. As shown in Fig. 1b,c, the proportion of MHC + nuclei increased from 1.29% on day 1 to 49.42% on day 4 (Fig. 1b), while the fusion index increased from 0.32% on day 1 to 43.13% on day 4 (Fig. 1c), representing a clear differentiation of C2C12 myoblasts into myotubes after four days.

**Figure 2:**
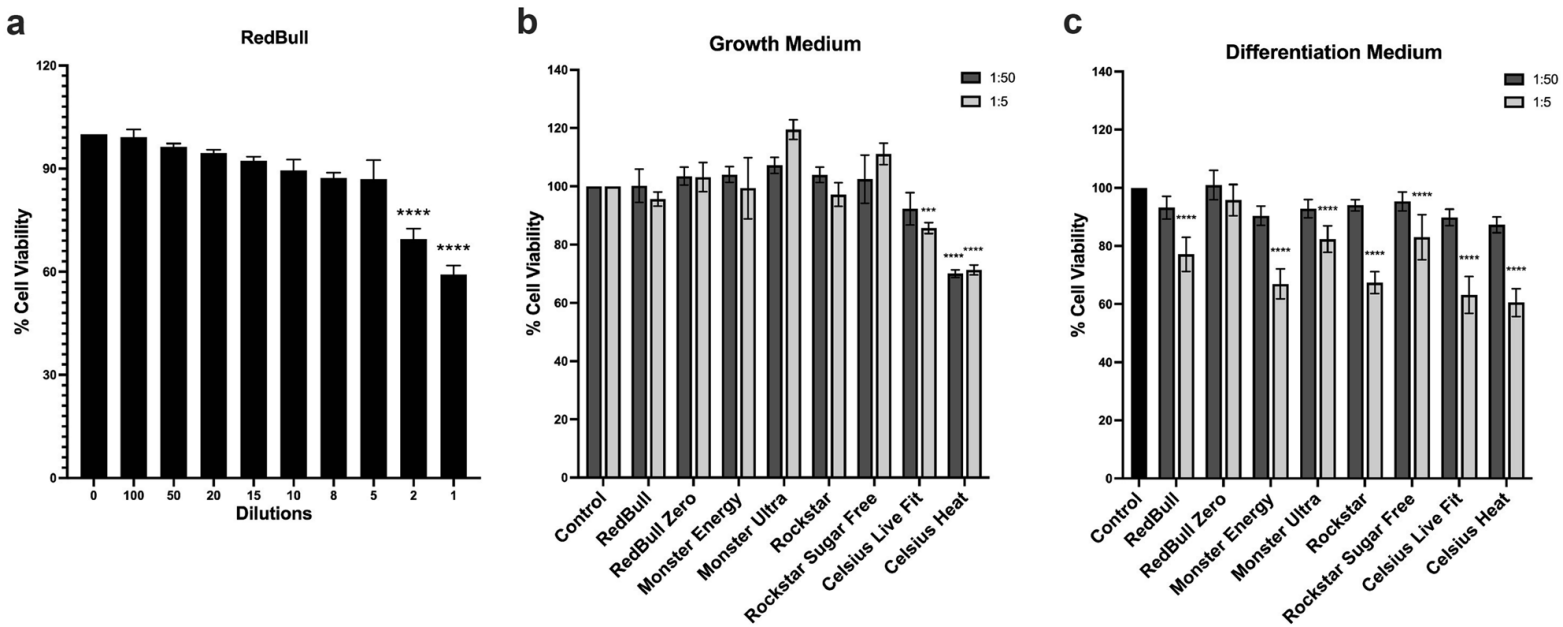
Effect of energy drinks on C2C12 cell viability. (**a**) Quantification of cytotoxicity in cells treated with 1:100, 1:50, 1:20, 1:15, 1:10, 1:8, 1:5, 1:2, and 1:1 dilutions of RedBull for 24 h. (**b**) Quantification of cytotoxicity in cells treated with 1:50 and 1:5 dilutions of all energy drinks including RedBull, RedBull Zero, Monster Energy, Monster Ultra Paradise, Rockstar, Rockstar Sugar Free, Celsius Live Fit, and Celsius Heat in growth medium for 24 h. (**c**) Quantification of cytotoxicity in cells treated with 1:50 and 1:5 dilutions of all energy drinks in differentiation medium for 24 h. Cell viability was evaluated using the MTS assay and is represented as a percentage of the untreated control. Means ± SD from four replicate values are shown. An ordinary one-way ANOVA test was used to test for significance compared to the untreated control (***p < 0.001, ****p < 0.0001).

To assess the possible cytotoxicity of the energy drinks and to determine the appropriate dilutions for subsequent myogenic differentiation assay, C2C12 cells were exposed to various dilutions of RedBull in growth medium for 24 h and analyzed for cell viability using an MTS assay. As shown in Fig. 2a, a majority of the dilutions did not impose a strong toxicity on cultured C2C12 cells with the exception of the 1:2 and 1:1 dilution which elicited 30% and 40% cell death, respectively. The dilutions 1:50 (the dose that barely cause any cell death) and 1:5 (the dose closest to the 1:2 dilution with minimal toxicity) were selected for further analysis. The subsequent MTS assays were performed to examine the effects of all energy drinks on cell viability in either growth medium or differentiation medium. As shown in Fig. 2b, most of the energy drinks at 1:50 and 1:5 dilutions did not elicit a cytotoxic response in growth medium except for Celsius Live Fit and Celsius Heat which showed 14% and 28% cell death at 1:5 dilutions, respectively. Interestingly, while the energy drinks at 1:50 dilution in differentiation medium did not cause any significant cell death, most of them at 1:5 dilution induced about 20–30% cell death in differentiation medium, with the exception of RedBull Zero displaying no significant difference between 1:50 and 1:5 dilution (Fig. 2c). Thus, these two dosages were used in the subsequent analysis of myogenic differentiation, and the final concentrations of each gradient in 1:50 and 1:5 dilutions were summarized in Supplementary Table 1.

### Treatment with energy drinks inhibit myogenic differentiation

Next, we investigated the effect of energy drinks on myogenic differentiation. C2C12 myoblasts were induced to differentiate into myotubes in the presence of eight different energy drinks, each at two different dilutions (1:50 and 1:5). Cells without exposure to any energy drink was used as a negative control. Myogenic differentiation was evaluated by the ratio of MHC + nuclei and the fusion index after four days of induction. As shown in Table 2, cells treated with energy drinks exhibited a dose-dependent decrease in the MHC + ratio compared to the control. Among the energy drinks, Celsius Live Fit and Celsius Heat revealed the strongest inhibitory effect with barely any MHC + nuclei can be seen at a 1:5 dilution. Furthermore, exposure to energy drinks elicited a more drastic effect on myoblast fusion. Six out of eight energy drinks at a 1:5 dilution, with the exception of RedBull and RedBull Zero, almost completely abolished any myotube formation (Table 2). Moreover, relative mRNA expression of two important muscle markers myogenin (MyoG) and muscle creatine kinase (MCK) were significantly reduced in cells exposed to energy drinks compared to the control. A majority of the energy drinks exhibited a reduction in relative MCK expression at a 1:5 dilution except for RedBull Zero and Rockstar Sugar Free. Taken together, these results indicate that all energy drinks have an inhibitory effect on C2C12 myogenic differentiation with some variation between the beverages. The subsequent paragraphs describe the results in further detail based on each energy drink brand.

**Table 2:** Summary of differentiation and fusion indexes.

Control	MHC + cells		Fusion index (%)	
	49.42		43.13	
Dilution	1:50	1:5	1:50	1:5
RedBull	24.47	16.09	16.15	6.15
RedBull Zero	21.45	17.68	13.04	11.62
Monster Energy	29.05	9.031	16.90	0.07
Monster Ultra	29.17	10.95	17.84	0.11
Rockstar	27.09	12.03	19.37	0.07
Rockstar SugarFfree	24.60	12.66	15.58	0.00
Celsius Live Fit	15.65	0.40	10.73	0.00
Celsius Heat	18.27	2.39	12.79	0.25

#### RedBull and RedBull Zero

**Figure 3:**
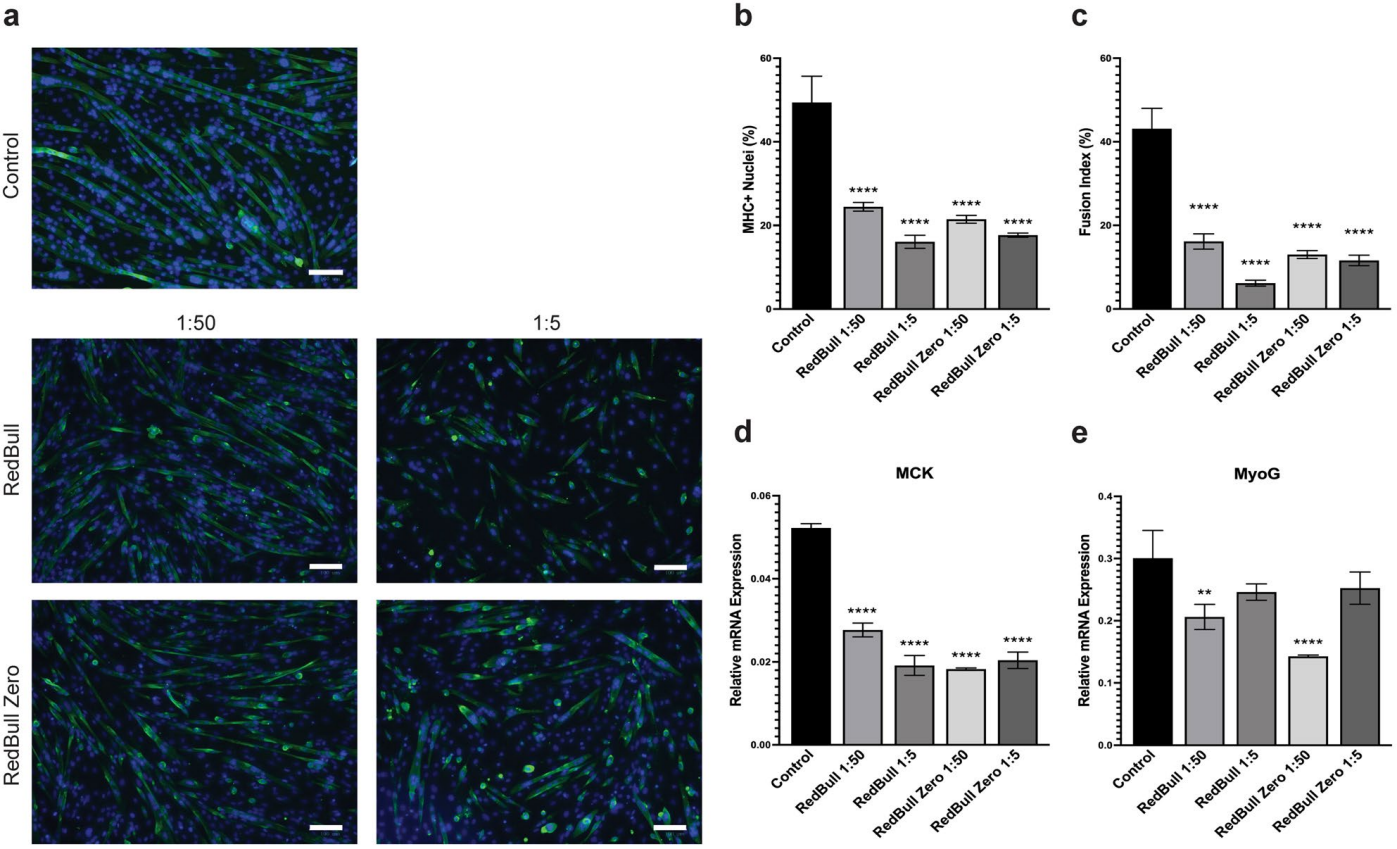
RedBull and RedBull Zero reduces C2C12 myogenic differentiation and myogenesis-related gene expression. (**a**) Representative images of C2C12 cells differentiated for 4 days with RedBull and RedBull Zero treatment at 1:50 and 1:5 dilutions. On day 4, cells were assessed for MHC + nuclei and fusion index by IF staining. Differentiated myotubes were stained with MF20 (green) and nuclei were stained with DAPI (blue). Images are representative of 2 different experiments. Scale bar = 100 µm. Quantification of the percentage of MHC + nuclei (**b**) and fusion index (**c**) in C2C12 cells differentiated for four days after RedBull and RedBull Zero treatment at 1:50 and 1:5 dilutions. Data is represented as means ± SD from four replicate images. Relative mRNA expression of MCK (**d**) and MyoG (**e**) in cells differentiated for four days under the same treatment conditions. Data is represented as means ± SD from triplicate values. All significance compared to the untreated controls in panels (**b**)–(**e**) were evaluated using a one-way ANOVA test (*p < 0.05, **p < 0.005, ***p < 0.001, ****p < 0.0001).

The effects of RedBull and RedBull Zero on C2C12 myogenic differentiation are shown in Fig. 3. The percentages of MHC + nuclei were significantly reduced for both RedBull and RedBull Zero (Fig. 3b; Table 2). Compared to about 49.42% MHC + nuclei in untreated controls, cells exposed to 1:50 and 1:5 dilutions of RedBull had only 24.47% and 16.09% MHC + nuclei, respectively (Fig. 3a,b; Table 2). Treatment with RedBull Zero yielded similar results, with 21.45% MHC + nuclei for 1:50 dilution and 17.68% for 1:5 dilution (Fig. 3a,b). Between the two energy drinks, RedBull appears to have a stronger inhibitory effect at higher concentration on myotube formation compared to RedBull Zero as evidenced by the lower fusion index (Fig. 3c). At a 1:5 dilution, the fusion indexes were 6.152% for RedBull and 11.62% for RedBull Zero, compared to 43.13% for the untreated control (Fig. 3c; Table 2). Furthermore, mRNA expression of myogenic differentiation marker, MCK, was significantly reduced in cells exposed to RedBull and RedBull Zero at both dilutions (Fig. 3d). Also, mRNA expression of MyoG was significantly reduced in cells exposed to 1:50 dilution of both energy drinks. However, cells exposed to a 1:5 dilution also showed a significant decrease but to a lesser extent (Fig. 3e). Thus, exposure to both RedBull and RedBull Zero elicited a decreased MCK and MyoG expression, but there appears to be no remarkable dose-dependence between the dilutions.

#### Monster energy and Monster Ultra Paradise

**Figure 4:**
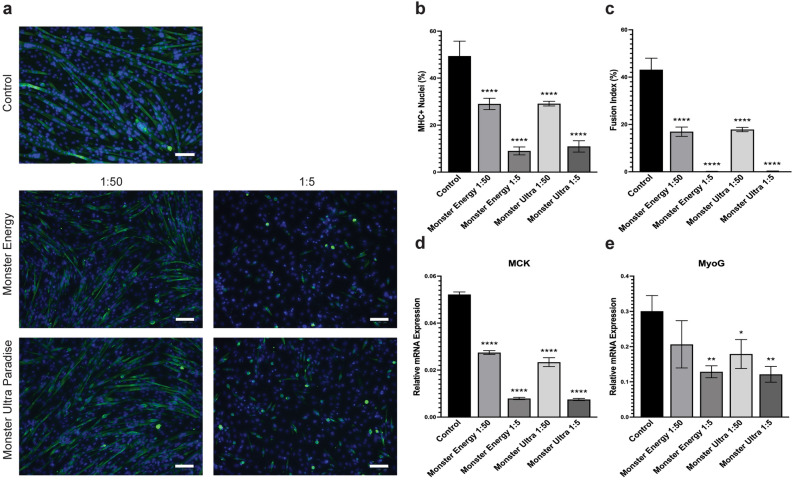
Monster Energy and Monster Ultra Paradise reduces C2C12 myogenic differentiation and myogenesis-related gene expression. (**a**) Representative images of C2C12 cells differentiated for 4 days with Monster Energy and Monster Ultra Paradise treatment at 1:50 and 1:5 dilutions. Cells were assessed for MHC + nuclei and fusion index by IF staining after 4 days of differentiation. Differentiated myotubes were stained with MF20 (green) and nuclei were stained with DAPI (blue). Images are representative of 2 different experiments. Scale bar = 100 µm. Quantification of the percentage of MHC + nuclei (**b**) and fusion index (**c**) in C2C12 cells differentiated for four days after Monster Energy and Monster Ultra Paradise treatment at 1:50 and 1:5 dilutions. Bars are represented as means ± SD from four replicate images. Relative mRNA expression of MCK (**d**) and MyoG (**e**) in cells differentiated for four days under the same treatment conditions. Data is represented as means ± SD from triplicate values. All significance compared to the untreated controls in panels B-E were evaluated using a one-way ANOVA test (*p < 0.05, **p < 0.005, ***p < 0.001, ****p < 0.0001).

The effects of Monster Energy and Monster Ultra Paradise on C2C12 myogenic differentiation are summarized in Fig. 4. A significant inhibition of myogenic differentiation and myotube formation was observed for both energy drinks and dilutions. Cells exposed to 1:50 and 1:5 dilutions of Monster Energy had about 29.05% and 9.03% MHC + nuclei, respectively. Similar results were found in cells exposed to 1:50 and 1:5 dilutions of Monster Ultra Paradise with 29.17% and 10.95% MHC + nuclei, respectively (Fig. 4b; Table 2). Comparing the two drinks, Monster Energy and Monster Ultra Paradise do not appear to have a difference in their inhibitory effects on myotube formation. Cells exposed to Monster Ultra Paradise had a fusion index of 17.84% and 0.11% when treated at a 1:50 and 1:5 dilution, respectively. Likewise, the indices were 16.90% and 0.07% when exposed to Monster Energy at a 1:50 and 1:5 dilution, respectively (Fig. 4c; Table 2). It is worth noting that Monster Energy and Monster Ultra Paradise displayed a much stronger inhibitory effect on myogenic differentiation at 1:5 dilution, as evidenced by a remarkable decrease in the number of MHC + nuclei (Fig. 4a,b) as well as an almost diminished fusion index (Fig. 4c). Furthermore, mRNA expression of MCK and MyoG were reduced significantly upon Monster Energy and Monster Ultra Paradise treatment at both 1:50 and 1:5 dilutions compared to the control (Fig. 4d,e). Collectively, all dilutions of Monster Energy and Monster Ultra Paradise have an inhibitory effect on differentiation with the 1:5 dilution causing a more significant decrease in the parameters evaluated.

#### Rockstar and Rockstar Sugar Free

**Figure 5:**
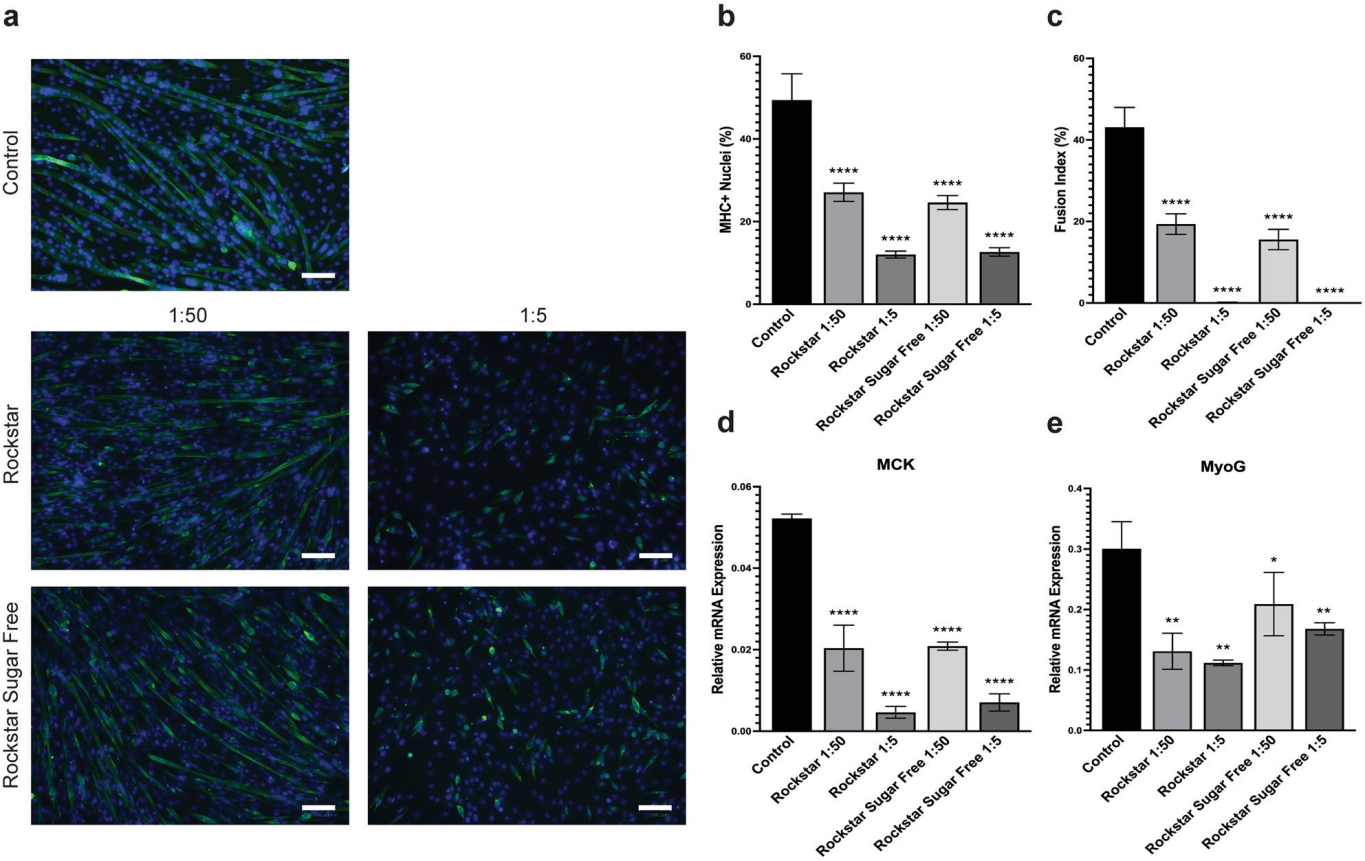
Rockstar and Rockstar Sugar Free reduces C2C12 myogenic differentiation and myogenesis-related gene expression. (**a**) Representative images of C2C12 cells differentiated for 4 days with Rockstar and Rockstar Sugar Free treatment at 1:50 and 1:5 dilutions. Cells were assessed for MHC + nuclei and fusion index by IF staining after 4 days of differentiation. Differentiated myotubes were stained with MF20 (green) and nuclei were stained with DAPI (blue). Images are representative of 2 different experiments. Scale bar = 100 µm. Quantification of the percentage of MHC + nuclei (**b**) and fusion index (**c**) in C2C12 cells after four days of differentiation with energy drink treatment at 1:50 and 1:5 dilutions. Bars are represented as means ± SD from four replicate images. Relative mRNA expression of MCK (**d**) and MyoG (**e**) in cells differentiated for four days under the same treatment conditions. Data is represented as means ± SD from triplicate values. All significance compared to the untreated controls in panels (**b**)–(**e**) were evaluated using a one-way ANOVA test (*p < 0.05, **p < 0.005, ***p < 0.001, ****p < 0.0001).

The effects of Rockstar and Rockstar Sugar Free on C2C12 myogenic differentiation are shown in Fig. 5. The myogenic differentiation and myotube formation were significantly inhibited by both Rockstar and Rockstar Sugar Free at 1:5 and 1:50 dilutions, similar to the level of inhibition seen by Monster energy drink treatments. Cells exposed to 1:50 and 1:5 dilutions of Rockstar had about 27.09% and 12.03% MHC + nuclei, respectively. Treatment with Rockstar Sugar Free yielded about 24.6% MHC + nuclei for 1:50 dilution and 12.66% for 1:5 dilution (Fig. 5a,b). The fusion indices were 19.37% and 15.58% for 1:50 dilution of Rockstar and Rockstar Sugar Free, respectively. Additionally, exposure to both energy drinks at a 1:5 dilution completely abolished the formation of multinucleated myotubes (Fig. 5a,c; Table 2). Relative MCK and MyoG mRNA expression levels were significantly reduced with Rockstar and Rockstar Sugar Free treatment in a dose-dependent manner (Fig. 5d,e). Interestingly, Rockstar Sugar Free appears have a less inhibitory effect than Rockstar at all dilutions for myogenic markers MCK and MyoG (Fig. 5d,e). Together, exposure to Rockstar and Rockstar Sugar Free elicited a significant decrease in myogenic differentiation, with a stronger inhibitory effect on myoblast fusion at a higher concentration.

#### Celsius Live Fit and Celsius heat

**Figure 6:**
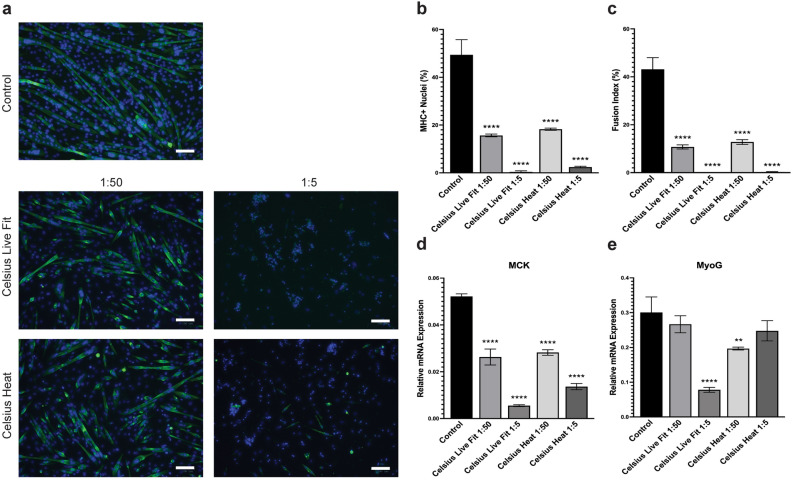
Celsius Live Fit and Celsius Heat reduces C2C12 myogenic differentiation and myogenesis-related gene expression. (**a**) Representative images of C2C12 cells differentiated for 4 days with Celsius Live Fit and Celsius Heat treatment at 1:50 and 1:5 dilutions. Cells were evaluated for MHC + nuclei and fusion index through IF staining after 4 days of differentiation. Differentiated myotubes were stained with MF20 (green) and nuclei were stained with DAPI (blue). Images are representative of 2 different experiments. Scale bar = 100 µm. Quantification of the percentage of MHC + nuclei (**b**) and fusion index (**c**) in C2C12 cells after four days of differentiation with energy drink treatment at 1:50 and 1:5 dilutions. Bars are represented as means ± SD from four replicate images. Relative mRNA expression of myogenic differentiation markers MCK (**d**) and MyoG (**e**) in cells differentiated for four days under the same treatment conditions. Data is represented as means ± SD from triplicate values. All significance compared to the untreated controls in panels (**b**)–(**e**) were evaluated using a one-way ANOVA test (*p < 0.05, **p < 0.005, ***p < 0.001, ****p < 0.0001).

The effects of Celsius Live Fit and Celsius Heat on C2C12 myogenic differentiation are summarized in Fig. 6. Comparable to Monster and Rockstar, both Celsius Live Fit and Celsius Heat at 1:50 and 1:5 dilutions significantly inhibited myogenic differentiation and myotube formation. Cells exposed to Celsius Live Fit at a 1:50 and 1:5 dilution had MHC + nuclei of 15.65% and 0.40%, respectively. Similarly, treatment with Celsius Heat at a 1:50 and 1:5 dilution had MHC + nuclei of 18.27% and 2.39%, respectively (Fig. 6b; Table 2). Fusion indices for Celsius Live Fit and Celsius Heat were 10.73% and 12.79%, respectively, at a 1:50 dilution but these were diminished to 0.00% and 0.25% at a 1:5 dilution (Fig. 6c; Table 2). In addition, both energy drinks induced a dose dependent decrease of MCK mRNA expression levels (Fig. 6d). Interestingly, the MyoG expression for Celsius Live Fit at a 1:5 dilution was significantly reduced compared to that at a 1:50 dilution but expression for Celsius Heat at a 1:5 dilution was greater compared to that at a 1:50 dilution (Fig. 6e). Taken together, exposure to a greater concentration of both energy drinks elicits a significant decrease in differentiation compared to the control with no difference between the two drinks.

### Energy drinks induce minimal myotube atrophy

**Figure 7:**
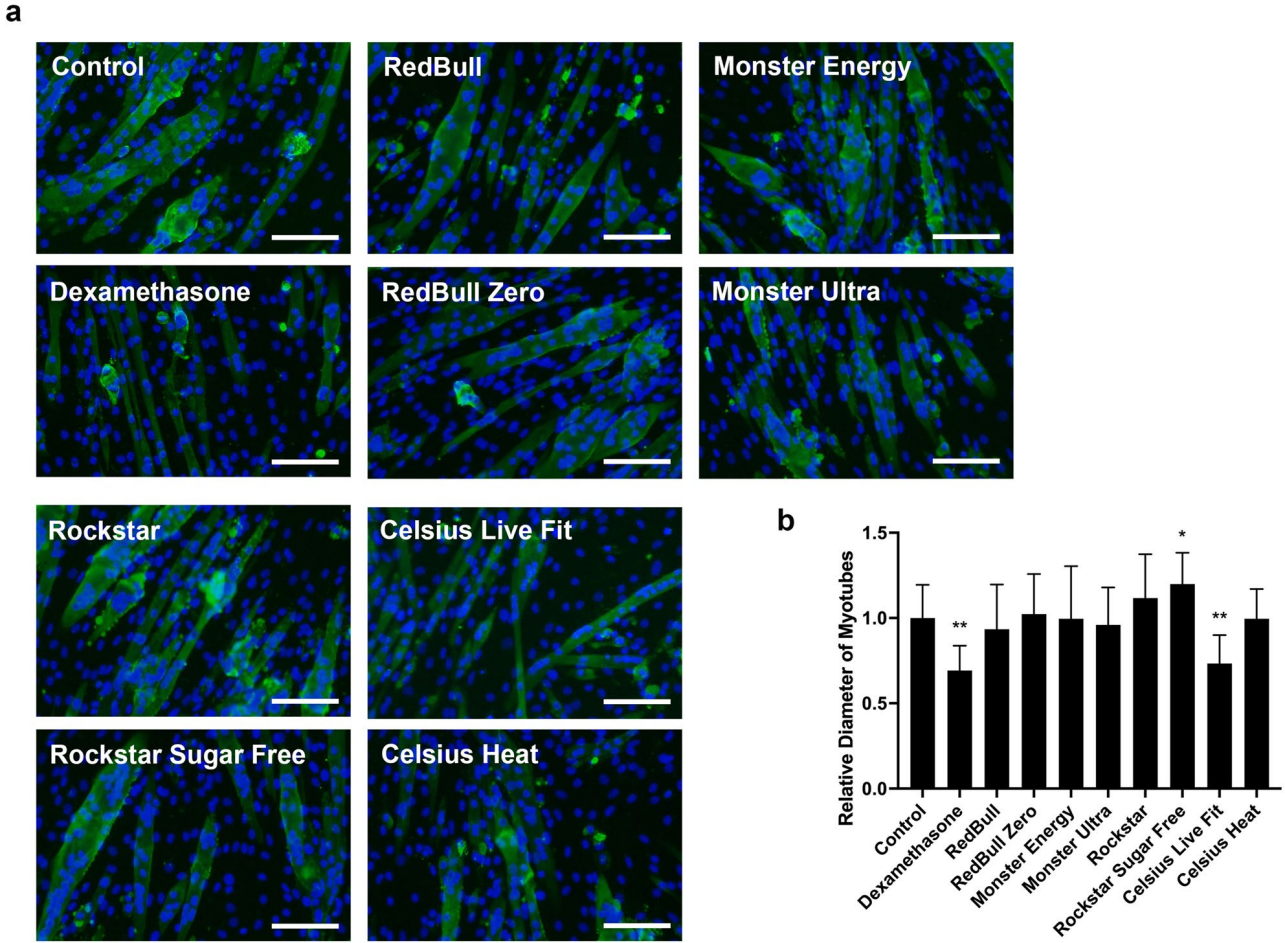
Effect of energy drinks on C2C12 myotube atrophy. C2C12 cells were differentiated for 4 days and followed by the treatment of either 10 μM dexamethasone or various energy drinks at 1:50 dilution in differentiation medium for 2 days. (**a**) Represented images showing the size of myotubes by immunofluorescence staining of MF20 (green) and DAPI (blue). Scale bar = 100 pixels. (**b**) Myotube diameter was assessed in myotubes with more than 10 nuclei, and presented as relative diameter of myotubes versus untreated controls. Data is represented as means ± SD (n > 10) (*p < 0.05, **p < 0.005).

To explore whether the energy drinks affect myotube atrophy, C2C12 cells were induced to differentiate to multinucleated myotubes. The myotubes were then treated with different energy drinks at a 1:50 dilution for 48 h. Dexamethasone (DEX), a glucocorticoid known to cause muscle atrophy, was used as a positive control^28,29^. As shown in Fig. 7, treatment with 10 uM of DEX for two days resulted in a significant decrease in the diameter of myotubes, as compared to the untreated controls. Most of the energy drinks at a 1:50 dilution did not induce significant change in myotube diameters (Fig. 7a,b), except for Celsius Live Fit and Rockstar Sugar Free. Interestingly, Rockstar Sugar Free induced a slight increase in myotube diameter (p < 0.05) but Celsius Live Fit exhibited a remarkable decrease in myotube diameter (p < 0.005), which is also comparable to those after DEX treatment (Fig. 7a,b). These results indicate that most of the energy drinks have minimal effects on myotube atrophy.

## Discussion

Energy drink brands aggressively market their drinks to enhance physical stamina, cognitive abilities, focus, and wakefulness—characteristics that appeal the most to young adults, students, athletes, and even military personnel^26^. Recent statistics show that the United States consumed the most caffeinated energy drinks per capita than any other country, especially prevalent among males between the ages of 18 and 34^26^. In addition to caffeine, energy drinks contain high concentrations of other ingredients such as taurine, B-vitamins, sucrose, glucuronolactone, sucrose, and herbal extracts^26,30^. As mentioned previously, this is a major public health concern because energy drink consumption has been shown to be associated with several fatal outcomes related to the cardiovascular system such as myocardial infarctions, cardiomyopathies, arrhythmias, and even sudden cardiac death^6,26^. Furthermore, there are increasing studies reporting the adverse health effects of energy drink consumption on multiple organ systems including the renal, endocrine, gastrointestinal, psychiatric, and neurological systems^26,31–33^. More specifically, there have been multiple case reports of chest tightness, chest pain, high blood pressure, arrhythmia, tachycardia, irritability, panic attacks, hallucinations, nervousness or anxiety, and epileptic seizures^30^. Despite these findings, there is still a huge gap of knowledge on the safety of energy drink consumption due to the lack of research and regulation on these beverages and their ingredients^26^. Given that there are limited studies observing the effect of energy drinks as a whole, this study aimed to compare the in vitro effects of energy drinks on the growth and differentiation of myoblast cells.

We first evaluated the cytotoxicity of C2C12 cells in response to varying dilutions of RedBull, and found that only two of the highest doses (1:1 and 1:2 dilutions) induced about 30–40% cell death (Fig. 1a). The 1:50 and 1:5 dilutions were selected for subsequent cytotoxicity analysis of eight different energy drinks, including RedBull, RedBull Zero, Monster Energy, Monster Ultra Paradise, Rockstar, Rockstar Sugar Free, Celsius Live Fit, and Celsius Heat. Our results showed that Celsius Live Fit and Celsius Heat at 1:5 dilutions in growth medium significantly decreased the number of viable cells, while other energy drinks at both 1:5 and 1:50 dilutions elicited very minimal cell death (Fig. 2b). On the contrary, all energy drinks except for RedBull Zero at a 1:5 dilution in differentiation medium elicited significant cell death compared to their respective 1:50 dilutions (Fig. 2c). It is evident that cell viability in response to energy drinks is different in growth or differentiation medium. Despite the differences, cell viability upon Celsius Live Fit and Celsius Heat exposure in differentiation medium shows consistent results with the myogenic differentiation findings where the 1:5 dilution exhibits less cells compared to the 1:50 dilution (Fig. 6a). Although it is obscure as to why Celsius Live Fit and Celsius Heat displayed a relatively higher cytotoxicity compared to other energy drinks, one potential explanation may be due to the high concentration of caffeine in Celsius drinks. Compared to an average caffeine concentration of 32.51 mg/100 ml among the other six drinks, Celsius Live Fit and Celsius Heat contain double the amount of caffeine at approximately 56.34 and 63.42 mg/100 ml (~ 3 mM), respectively (Table 1). The final caffeine concentrations used in the differentiation medium are approximately 58 μM for Celsius Live Fit and 65 μM for Celsius Heat at 1:50 dilutions (Supplementary Table 1). Previous studies have indicated that the physiological concentration of caffeine is usually less than 70 μM and plasma concentrations range between 20 and 50 μM^34,35^. Therefore, the doses selected for this study are of physiological relevance. Caffeine has been previously shown to elicit cytotoxicity in multiple cell types. It was reported that caffeine displayed a strong cytotoxic effect on cultured rat heart cells at 20 mM^36^ and on RT-2 glioma cells at 1 mM^37^. In addition, MCF-7 breast cancer cells showed cytotoxicity at as low as 80 μM of caffeine^38^. Consistently, our study shows significant cytotoxicity in C2C12 cells treated with a 1:5 dilution of Celsius Live Fit and Celsius Heat (approximately 0.6 mM caffeine). A future study with double the amount of caffeine in other energy drinks, such as RedBull, Monster, or Rockstar brands, will likely address whether the high concentration of caffeine in Celsius Live Fit and Celsius Heat accounted for the cytotoxicity in C2C12 cells. Additionally, it is worth noting that Celsius Live Fit and Celsius Heat are the only energy drinks containing chromium chelate, a form of chromium (i.e. chromium picolinate, chromium chloride, chromium histidinate, chromium nicotinate, and chromium polynicotinate) that is often found in dietary supplements due to their reported function in reducing risk of type 2 diabetes^39^ and facilitating weight loss^40^. However, there is also conflicting evidence reporting potential toxicity of chromium as a dietary supplement such as renal impairment^41^ and formation of oxidative damage^42^.

Our results showed a dose-dependent inhibition of C2C12 myogenic differentiation for all energy drink treatments. Upon induction, myoblasts undergo differentiation into muscle fibers—a process that is orchestrated by myogenic regulatory factors MyoD, MyoG, Myf5, Myf6. A critical step in myogenic differentiation involves the fusion of myoblasts to produce multi-nucleated myotubes. Generally, MHC + nuclei represent differentiated myocytes and the fusion index represents fused multi-nucleated myotubes. In our study, all energy drinks at a higher concentration exhibited decreased ratio of MHC + nuclei, fusion index and expression of myogenic differentiation markers, suggesting that inhibition of myogenesis is a common effect across all energy drinks. Again, caffeine may be a potential ingredient contributing to this inhibition. Supportively, a recent study reported that caffeine treatment results in reduced mRNA expression of myogenesis-related genes such as MYH7B, MEF2A, Myod1, MyoG, and Pax7 in developing chicken embryos^43^. Moreover, treating fully differentiated C2C12 myotubes with high dosages of caffeine (2.5–10 mM) induced autophagy^44^, while treating with lower concentration of caffeine (0.5 mM) led to increased mitochondrial turnover and fatty acid oxidation^45^. An additional study showed that incubation of differentiated C2C12 myotubes with more than 1 mM caffeine for 24 h significantly reduced the size of myotubes and the protein content^46^. Taken together, caffeine treatment not only inhibited myogenic differentiation but also impaired function of fully differentiated myotubes. Consistent with this notion, our study showed that Celsius Live Fit and Celsius Heat, containing double the amount of caffeine compared to other energy drinks, displayed the highest capacity to inhibit myogenic differentiation (Fig. 6; Table 1). Moreover, exposure of differentiated myotubes to Celsius Live Fit induced a significant decrease in myotube diameters, which is similar to the myotube atrophy induced by dexamethasone (Fig. 7). This suggests that the treatment of energy drinks may impact myotube functions. Interestingly, Celsius Live Fit contains a high concentration of caffeine compared to other energy drinks but still is less than that in Celsius Heat. It is yet unclear as to which ingredient in Celsius drinks is responsible for this variation but caffeine is likely to be a contributing factor. Previous studies have shown that acute exposure to a high concentration of caffeine reduced myotube diameter possibly through stimulation of the ubiquitin–proteasome system or inhibition of the Akt/mTOR/p70S6K signaling pathway^46,47^. Despite the notion that caffeine may have ergogenic benefits among athletes^48–50^, several studies have demonstrated negative effects associated with caffeine consumption^43,51,52^. Acute ingestion of caffeine has been shown to cause an increase in blood pressure, which can then lead to further cardiovascular complications such as strokes or heart failures^51^. Another main ingredient in energy drinks is taurine, an essential or a semi-essential amino acid. Taurine has been suggested to have protective effects on C2C12 myoblasts with impaired differentiation properties^53^. A different study also reported similar findings where gossypol-induced apoptosis was attenuated by taurine in C2C12 mouse myoblasts, possibly through the GPR87-AMPK/AKT signaling pathway^54^. Taurine has also been shown to enhance iron-related proteins as well as reduce lipid peroxidation in differentiated C2C12 myotubes^55^. All energy drinks included in this study contained taurine but the amount of taurine in each formula was not specified. Therefore, it is impossible to conclude if taurine displayed any protective effect on myogenic differentiation.

Interestingly, RedBull and RedBull Zero exhibited a relatively minor inhibitory effect on myocyte fusion compared to the other drinks (Table 2). A potential ingredient that may be responsible for this difference is vitamin B6 because there is approximately double the amount of vitamin B6 in RedBull and RedBull Zero (Table 1). One study reported that the vitamin B6 metabolism pathway was upregulated during differentiation of primary human skeletal muscle cells^56^. Vitamin B6’s active form, pyridoxal phosphate, is a coenzyme essential for one carbon metabolism during myogenic progression^56^. Another study also reported the importance of vitamin B6 in myogenesis as mice with a vitamin B6 deficient diet showed a decreased number of quiescent satellite cells^57^. According to several studies, vitamin B6 appears to have a beneficial effect on myogenic progression and differentiation but it is yet unknown whether this ingredient is the cause of the relatively lower inhibitory effect of RedBull and RedBull Zero on the fusion index. Further studies should be performed to investigate the exact role of vitamin B6 on C2C12 muscle differentiation.

There are several limitations to this study. One limitation is that the energy drinks were tested using only the C2C12 cell line, which is a well-accepted model to study myoblast differentiation but further studies should consider in vivo experiments as well as primary murine skeletal muscle cells. Another limitation is that the energy drinks dilutions were selected based on their cytotoxic effects on C2C12 cells so they may not be fully representative of human energy drink consumption rates. Moreover, this study does not provide mechanistic insight on specific molecules involved, as it aimed to compare the effect of whole energy drinks with various formulation. Further studies altering single ingredients to match the concentration in different energy drinks will help to identify key molecules that may inhibit myogenic differentiation and myotube function. Nevertheless, this is the first study to observe the in vitro effects of various energy drink formulations on the growth and differentiation of myoblasts. The findings suggest that all energy drinks—RedBull, RedBull Zero, Monster Energy, Monster Ultra Paradise, Rockstar, Rockstar Sugar Free, Celsius Live Fit, and Celsius Heat—inhibit C2C12 myogenic differentiation with some having a greater inhibitory effect than others. Accordingly, differences in their effects on myogenic differentiation may be attributable to variations in energy drink formulas so future studies should investigate single ingredients or a combination of ingredients associated with adverse health outcomes in order to establish FDA regulations on energy drinks.

## Methods

### Energy drink preparation

The eight energy drinks used in this study include RedBull, RedBull Zero, Monster Energy, Monster Ultra Paradise, Rockstar, Rockstar Sugar Free, Celsius Live Fit, and Celsius Heat. Respective ingredients and pH levels are listed in Table 1. The energy drinks were adjusted to a pH of approximately 7.0 using 1N NaOH and sterilized using a 0.22 µm syringe filter. The final concentrations of essential ingredients in differentiation media are shown in Supplementary Table 1.

### Cell culture and myogenic differentiation

The mouse myoblast C2C12 cells (American Type Culture Collection; Manassas, VA, USA) were cultured in high glucose Dulbecco’s modified Eagle growth medium (DMEM; Gibco, USA) supplemented with 20% fetal bovine serum (FBS; Gibco, USA) and 1% penicillin–streptomycin (Gibco, USA). To induce differentiation, cells were switched to differentiation media which consists of high glucose DMEM (Gibco, USA) supplemented with 2% horse serum (Thermo Fisher Scientific, USA) and 1% penicillin–streptomycin (Gibco, USA) for 4 days. The differentiation media was changed every two days.

For energy drink treatments, cells were seeded at a density of 100,000 cells/well in a 24-well plate and incubated at 37 °C under 5% CO_2_ for 24 h in growth media. Growth medium was then replaced with differentiation medium containing respective energy drinks. Cells were treated with either RedBull, RedBull Zero, Monster Energy, Monster Ultra Paradise, Rockstar, Rockstar Sugar Free, Celsius Live Fit, or Celsius Heat at 1:50 and 1:5 dilutions. Cells were differentiated for 4 days.

### Cell viability assay (MTS assay)

Cells were seeded at a density of 10,000 cells/well in a 96-well plate and incubated at 37 °C for 24 h in growth media. Cells were treated with 1:100, 1:50, 1:20, 1:15, 1:10, 1:8, 1:5, 1:2, and 1:1 dilution of RedBull in growth media. After 24 h in culture, CellTiter 96 AQueous One Solution Reagent (MTS Reagent; Promega, USA) was added to each well and incubated at 37 °C for 3 h. Absorbance was measured at 490 nm using a SpectraMax 340PC384 microplate reader (Marshall Scientific, USA). In a subsequent assay, the same protocol was used for cells treated with 1:50 and 1:5 dilutions for all energy drink brands in either growth or differentiation media.

### Immunofluorescence staining

Differentiated cells were washed with PBS 1X before being fixed in 10% formalin at room temperature for 10 min. The cells were washed with PBS followed by permeabilization with 0.1% Triton X-100 in PBS for 2 min. They were then washed with 0.1% Tween-20 in PBS (PBST) prior to being blocked with 5% bovine serum albumin (BSA; Thermo Fisher Scientific, USA) for 1 h. The cells were incubated overnight with myosin heavy chain (MHC) antibody (MF20; DSHB, USA) at a concentration of 3 µg/mL. The following day, cells were incubated in Alexa Fluor 488 goat anti-mouse IgG (H + L) cross-adsorbed secondary antibody (Thermo Fisher Scientific, USA) at a 1:2000 dilution. After washing with PBST, the cells were stained with 1X 4′,6-diamidino-2-phenylindole (DAPI; Thermo Fisher, USA) for 5 min and briefly washed with PBST before imaging. In order to assess muscle atrophy, C2C12 cells were differentiated for four days followed by treatment with either 10 uM dexamethasone or various energy drinks at 1:50 dilutions for an additional 48 h. The cells were imaged with a ZOE Fluorescent Cell Imager (Hercules, USA) and images were analyzed using ImageJ (National Institute of Health, USA).

### Quantitation of myogenic differentiation and muscle atrophy

Myogenic differentiation was assessed using the percentage of MHC + nuclei and the fusion index^58^. The MHC + nuclei value represents the proportion of MHC + nuclei out of the total number of nuclei. The fusion index represents the proportion of MHC + nuclei in fused myotube with 2 or more nuclei out of the total nuclei. Four images were quantified for each treatment condition and the average was used for statistical analysis. Muscle atrophy was quantified using ImageJ by measuring the maximum diameters of MHC + myotubes with more than 10 nuclei in three fields.

### Real-time quantitative PCR (RT-qPCR)

On day 4 of differentiation, cells were harvested for RNA extraction using Tri-reagent following the manufacturer’s protocol (Molecular Research Center, USA). RNA concentrations were quantified using a spectrophotometer (NanoDrop-2000; Thermo Fisher Scientific, USA) and complementary DNA (cDNA) was synthesized using the LunaScript RT Enzyme Supermix Kit (New England Biolabs NEB; MA, USA) according to the manufacturer’s protocol. Real-time quantitative PCR was performed on a QuantStudio 7 Flex Real-Time PCR (Thermo Fisher Scientific, USA) at a final volume of 10 uL using PowerUp SYBR Green Master Mix (Applied Biosystems, USA). The mouse primers used include MCK (forward primer 5′–3′: AACCTCAAGGGTGGAGACGA; reverse primer 5′–3′: GTGCGGAGGCAGAGTGTAAC; Thermo Fisher Scientific, USA), MyoG (forward primer 5′–3′: TCCAGAGAGCCCCCTTGTTA; reverse primer 5′–3′: GGTCAGGGCACTCATGTCTC; Thermo Fisher Scientific, USA), and reference gene *β*-actin (forward primer 5′–3′: ACCAGTTCGCCATGGATGAC; reverse primer 5′–3′: TGCCGGAGCCGTTGTC; Thermo Fisher Scientific, USA). Amplifications were done in triplicates for all target and housekeeping genes. The raw threshold cycle (Ct) values were used to calculate ΔΔCt values, which represent relative mRNA expression levels of each target gene.

### Statistical analysis

ordinary one-way ANOVA test (Prism version 9; GraphPad, USA) was used to determine the significance of energy drink dilutions compared to the control for all parameters investigated. Data are all represented as mean ± SD and significance is classified as *p < 0.05, **p < 0.005, ***p < 0.001, ****p < 0.0001.

## Supplementary Information

Supplementary Table S1.

## Data Availability

The datasets used and/or analyzed during the current study are available from the corresponding author on reasonable request.
