# Supplementary material for: Effects of energy drinks on myogenic differentiation of murine C2C12 myoblasts
Source: Sci Rep. 2023 May 25;13:8481. doi: 10.1038/s41598-023-35338-7 (PMC10213057; doi:10.1038/s41598-023-35338-7)
Supplement: Supplementary file 1 — Supplementary Table S1. [file 41598_2023_35338_MOESM1_ESM.pdf]

Effects of energy drinks on myogenic differentiation of murine C2C12 myoblasts

Sun Young Park, Georgia Karantenislis, Hannah T Rosen, Hong Sun*

Supplementary Table 1. Energy Drink Ingredient Table Concentration

	RedBull 1:50	RedBull 1:5	RedBull Zero 1:50	RedBull Zero 1:5	Monster Energy 1:50	Monster Energy 1:5	Monster Ultra 1:50	Monster Ultra 1:5
Caffeine (μM)	32.96	329.58	32.96	329.58	34.84	348.4	30.484	304.84
Sodium (mM)	365.38	3653.8	104.39	1043.94	680.51	6805.12	570.16	5701.58
Calcium (μM)	59.884	598.84	59.884	598.84	-	-	-	-
Riboflavin or B2 (μM)	-	-	-	-	379.96	3799.56	-	-
Niacin or B3 (mM)	10.40	103.97	10.40	103.97	13.74	137.39	13.74	137.39
Pantothenic Acid or B5 (μM)	912.28	9,122.84	912.28	9,122.84	-	-	3,857.14	38,571.36
Vitamin B6 (μM)	2,009.70	20,096.94	2,009.70	20,096.94	1,020.22	10,202.16	1,020.22	10,202.16
Vitamin B12 (μM)	113.33	1,133.26	113.33	1,133.26	374.36	3,743.60	352.08	3,520.78
Vitamin C (μM)	-	-	-	-	-	-	-	-
L-citrulline (μM)	-	-	-	-	-	-	-	-

Supplementary Table 1 cont. Energy Drink Ingredient Table Concentration

	Rockstar 1:50	Rockstar 1:5	Rockstar Sugar Free 1:50	Rockstar Sugar Free 1:5	Celsius Live Fit 1:50	Celsius Live Fit 1:5	Celsius Heat 1:50	Celsius Heat 1:5
Caffeine (μM)	34.84	348.4	34.84	348.4	58.024	580.24	65.322	653.22
Sodium (mM)	128.74	1287.44	441.41	4414.14	0	0	0	0
Calcium (μM)	-	-	-	-	70.288	702.88	52.75	527.52
Riboflavin or B2 (μM)	146.14	1461.36	146.14	1461.36	254.54	2545.44	190.77	1907.74
Niacin or B3 (mM)	5.50	54.96	5.50	54.96	9.15	91.53	6.87	68.69
Pantothenic Acid or B5 (μM)	964.28	9,642.84	964.28	9,642.84	2,569.90	25,699.04	1,928.57	19,285.68
Vitamin B6 (μM)	424.40	4,244.00	424.40	4,244.00	665.56	6,655.64	500.06	5,000.60
Vitamin B12 (μM)	74.81	748.12	74.81	748.12	249.38	2,493.76	187.11	1,871.06
Vitamin C (μM)	-	-	-	-	19,193.60	191,936.00	14,404.95	144,049.52
L-citrulline (μM)	-	-	-	-	-	-	482.88	4,828.76
